# Association of aerobic and muscle-strengthening physical activity with chronic kidney disease in participants with hypertension

**DOI:** 10.1186/s40885-024-00291-8

**Published:** 2024-11-01

**Authors:** Yunmin Han, Younghwan Choi, Yeon Soo Kim

**Affiliations:** 1 Seoul National University, Seoul, Republic of Korea; 2 Institute of Sports Science, Seoul National University, Seoul, Republic of Korea

**Keywords:** Aerobic physical activity, Muscle strengthening exercise, Chronic kidney disease, Hypertension

## Abstract

**Background:**

In Korea, chronic kidney disease (CKD) is increasingly prevalent among adults with hypertension, of which approximately 30% of the population is affected. Despite the recognized benefits of adherence to physical activity (PA) recommendations, including aerobic and muscle-strengthening activities (MSA), the impact of such adherence on the prevalence of CKD in individuals with hypertension has not been extensively studied. This study aimed to investigate the association between aerobic PA and MSA levels, and the prevalence of CKD in individuals with hypertension.

**Methods:**

This study included 5,078 individuals with hypertension using data from the Korean National Health and Nutrition Examination Survey (2019–2021). PA levels were measured as min/week of moderate-to-vigorous PA (MVPA) based on self-reports, and MSA was quantified as the number of days per week. CKD was defined as an estimated glomerular filtration rate (eGFR) < 60 mL/min/1.73 m². Logistic regression analysis evaluated the association between meeting PA guidelines and CKD after adjusting for potential confounders. Additionally, a joint analysis was conducted to assess the combined effects of MVPA and MSA on CKD.

**Results:**

After adjusting for all covariates, higher MVPA was associated with a lower prevalence of CKD. Compared to the group with inactive, the group with MVPA 1–149 min/week had an odds ratio (OR) of 0.80 (95% confidence interval [CI], 0.61–1.05), the group that met the MVPA 150–299 min/week criteria had an OR of 0.85 (95% CI, 0.62–1.17), and the group that met the MVPA ≥ 300 min/week criteria had an OR of 0.53 (95% CI, 0.37–0.76). MSA alone did not show a significant association with CKD. In the joint analysis, the group that met the MVPA and MSA guidelines had the lowest OR of 0.54 (95% CI, 0.34–0.86), compared to the group that did not meet either.

**Conclusions:**

MVPA was associated with the prevalence of CKD in participants with hypertension but not in those with MSA alone. However, compared with the group that did not meet both guidelines, the group that met both guidelines showed the lowest prevalence of CKD.

## Introduction

Chronic kidney disease (CKD) is a significant global public health concern affecting > 10% of the global population [1]. Recently, the prevalence of CKD has been increasing, with a significant increase in non-communicable diseases, such as hypertension, being a major contributing factor [2].

Hypertension is a major public health concern, with a global prevalence that has more than doubled over the past 20 years [3]. In Korea, the prevalence of hypertension in adults is approximately 30% [4]. Hypertension, characterized by constricted blood vessels and disturbances in renal function, including impaired sodium excretion, contributes to the rapid progression of chronic renal failure [5, 6]. Therefore, patients with chronic diseases should manage their lifestyles to prevent progression to more severe diseases. Regular aerobic physical activity (PA) can provide health benefits to patients with hypertension by improving arterial and metabolic function and reducing cardiovascular risk [7]. Additionally, some studies have shown that aerobic and resistance exercises can improve kidney function and lower blood pressure [8, 9]. The World Health Organization (WHO) PA guidelines recommend that individuals with chronic diseases engage in the same amount of PA as the general population, including at least 300 min/week of moderate-to-vigorous PA (MVPA) for additional benefits [10]. Additionally, the guidelines advise performing muscle-strengthening activities (MSA) for at least two days per week to further enhance health outcomes.

Observational studies have demonstrated that PA improves kidney function [11]. Integrating MVPA with MSA improves arterial and metabolic functions, lowers cardiovascular risks, enhances insulin sensitivity, regulates blood pressure, and improves kidney function [12, 13]. These synergistic effects can lead to better oxygenation of the kidney cells, strengthened vascular walls, and more effective waste removal.

Despite these known benefits, no study has yet investigated the association between aerobic PA, muscle strength activities, and the prevalence of CKD in patients with hypertension using national data. Therefore, this study aimed to conduct both independent and joint analyses to explore the association of aerobic PA and/or MSA with the prevalence of CKD.

## Methods

### Study participants

The Korea National Health and Nutritional Examination Survey (KNHANES) is an annual nationwide survey conducted by the Korean Disease Control and Prevention Agency. The survey collects data on Korean health status, behaviors, and nutrition and is used to monitor the health of the Korean population. Data were collected from the eighth cycle of the KNHANES, conducted between 2019 and 2021. The study population comprised adults (age ≥ 19) with hypertension, defined as having a systolic or diastolic blood pressure of ≥ 140 or ≥ 90 mmHg, respectively, or taking antihypertensive medication [14]. The analysis included 5,078 participants, after excluding invalid PA data and missing major covariates. This study was approved by the Institutional Review Board (IRB) of Seoul National University (IRB NO. E2311/001–003) and waived the requirement for informed consent because of the use of de-identified data.

### Assessment of physical activity and muscle-strengthening activity

PA was assessed using the Global Physical Activity Questionnaire (GPAQ), which measures the intensity, duration, and frequency of aerobic PA across three domains; work, transportation, and leisure [15]. The GPAQ asks participants to report the frequency per week, hours, and minutes they engage in PA lasting ≥ 10 min in each domain. Moderate PA (MPA) is defined as any activity that expends 4.0 metabolic equivalent of a task (MET), causing slight increases in breathing or heart rate. Vigorous PA (VPA) is defined as any activity that expends 8.0 MET, causing significant increases in breathing or heart rate. Considering the different PA intensities, the total PA (total MVPA in min/week) of participants who engaged in any MVPA was calculated by multiplying VPA by 2; total PA = MPA (min/week) + [2 × VPA (min/week)] [16]. The frequency of MSA (e.g., push-ups, sit-ups, dumbbells, or barbell exercises) was assessed by asking participants the frequency they performed any MSA weekly.

The WHO PA guidelines require individuals to engage in aerobic MPA, VPA, and combined MVPA for at least 150, 75, and 150 min/week, respectively, with additional health benefits associated with ≥ 300 min of MVPA weekly [17]. The WHO recommends that adults perform MSA for all major muscle groups ≥ 2 days/week. Therefore, aerobic PA was divided into groups of 0 min, 1–149 min, 150–299 min, and ≥ 300 min of MVPA. Additionally, MSA was analyzed by dividing it into groups of 0-1 days/week, 2–3 days/week, and ≥ 4 days/week. The variables that combined the PA guidelines were categorized into four groups; (1) insufficient aerobic PA and MSA recommended levels, (2) sufficient MSA and insufficient aerobic PA recommended levels, (3) sufficient aerobic PA and insufficient MSA recommended levels, and (4) sufficient aerobic PA and MSA recommended levels.

### Study outcomes

Serum creatinine levels were measured in participants who had fasted for ≥ 8 h and compensated for the Jaffé kinetic colorimetric method using a Cobas analyzer (Roche, Germany) in a professional blood test laboratory. CKD was defined as an estimated glomerular filtration rate (eGFR) of < 60 mL/min/1.73 m^2^, calculated using the new CKD-Epidemiology Collaboration equation. [18].

### Covariates

Sociodemographic covariates included age, sex (male and female), family income (low, mid-low, mid-high, or high), and educational level (high school, high school graduate, or college). Smoking status was assessed using self-reported current smoking status. Alcohol consumption was assessed by asking the participants the amount of alcoholic drinks they typically consume weekly. Heavy alcohol consumption was defined as consuming ≥ 7 drinks weekly for men and ≥ 5 drinks weekly for women. Blood pressure was measured thrice at 5-min intervals, and the average of the second and third measurements was used as the final value. Type 2 diabetes mellitus was defined by any of the following; fasting plasma glucose level ≥ 126 mg/dL, HbA1_C_ level ≥ 6.5%, and currently taking any antidiabetic medication [19]. Dyslipidemia was defined by any of the following; total cholesterol level ≥ 240 mg/dL, high low-density lipoprotein cholesterol level ≥ 160 mg/dL, low high-density lipoprotein cholesterol level < 40 mg/dL in men and < 50 mg/dL in women, elevated triglycerides level ≥ 200 mg/dL, and currently using any lipid-lowering medication [20].

### Statistical analyses

Baseline characteristics were presented as mean ± SD and proportions (%) for continuous and categorical variables, respectively. Differences between groups were assessed using a general linear model for continuous variables and the Chi-square test for categorical variables. The association among CKD, MVPA, and MSA levels was analyzed using logistic regression analysis to evaluate odds ratios (ORs) and 95% confidence intervals (CIs). The MET values were standardized, and logistic regression models were employed to estimate the ORs and 95% CIs for CKD prevalence associated with a 1SD increase in PA. To examine the potential confounding effects of the following covariates on the association of MVPA and MSA with CKD, we adjusted for them in our analysis; model 1; age and sex, model 2; model 1 + current smoking, alcohol consumption, education level, income level, BMI, systolic blood pressure (SBP), aerobic PA (if MSA was the main exposure), and MSA (if aerobic PA was the main exposure), and model 3; model 2 + diabetes and dyslipidemia. Additionally, a joint analysis was performed by categorization based on meeting the recommendations of the MVPA and MSA guidelines to confirm the ORs. The statistical significance level was set at *p* < 0.05 for two-sided tests. All statistical analyses were performed using Stata 17.0 (Stata-corp., College Station, TX, USA).

## Results

The baseline characteristics according to aerobic PA levels are presented in Table 1. A total of 5,078 participants (2,608 women) were included, with a mean age of 62.9 ± 12.8 years. The mean eGFR value was 85.9 ± 17.3 mL/min/1.73 m^2^, with a significant difference in eGFR values according to aerobic PA levels (*p* < 0.001).

**Fig. 1:**
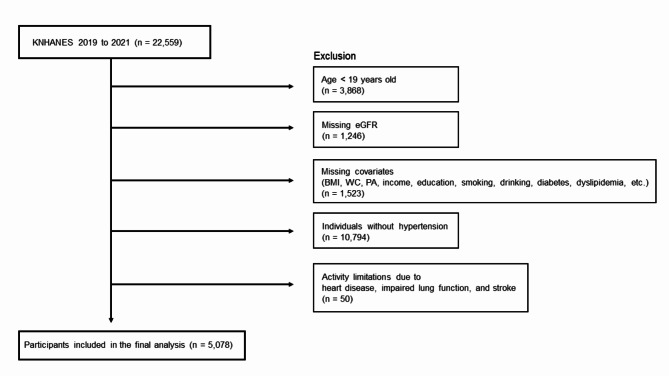
Flowchart of selecting participants for the study. KNHANES, Korea National Health and Nutritional Examination Survey; eGFR, estimated glomerular filtration rate; BMI, body mass index; WC, waist circumference; PA: physical activity

Figure 1 displays the exclusion criteria and the number of participants who were excluded from the study. Table 2 shows the OR with 95% CIs for CKD according to aerobic PA and MSA levels.

**Table 1:** Participants’ characteristics by physical activity levels

		Moderate-to-Vigorous Physical Activity (min/week)			*p*-value		
	Inactive (*n* = 2,077)	1–149 (*n* = 1,187)	150–299 (*n* = 835)	≥ 300 (*n* = 979)			
Female (n, %)	1,115 (53.7)	655 (55.2)	439 (52.8)	399 (40.7)	< 0.001		
Age (years)	65.5 ± 11.9	62.6 ± 12.9	61.6 ± 12.7	58.9 ± 13.4	< 0.001		
Height (cm)	160.2 ± 9.5	161.4 ± 9.6	162.1 ± 9.1	164.6 ± 9.5	0.398		
Weight (kg)	65.3 ± 12.7	66.2 ± 13.5	66.9 ± 13.4	69.1 ± 14.0	0.003		
Education level (n, %)					< 0.001		
< High school	1,233 (59.4)	557 (46.9)	363 (43.5)	319 (32.6)			
High school graduate	517 (24.9)	376 (31.7)	233 (27.9)	343 (35.0)			
≥ college	327 (15.7)	254 (21.4)	239 (28.6)	317 (32.4)			
Income level (n, %)					< 0.001		
Low	730 (51.6)	332 (28.0)	201 (24.1)	195 (19.9)			
Mid-Low	550 (26.5)	339 (28.6)	223 (26.7)	244 (24.9)			
Mid-High	450 (21.7)	277 (23.3)	197 (23.6)	249 (25.4)			
High	347 (16.7)	239 (20.1)	214 (25.6)	291 (29.7)			
Body Mass Index (kg/m^2^)	25.3 ± 3.6	25.3 ± 3.7	25.3 ± 3.7	25.4 ± 3.7	0.848		
Current Smoking (n, %)	335 (16.1)	167 (14.1)	123 (14.7)	159 (16.2)	0.352		
Heavy alcohol consumption (n, %)	253 (12.2)	139 (11.7)	111 (13.3)	162 (16.6)	0.003		
Diabetes (n, %)	648 (31.2)	318 (26.8)	229 (27.4)	265 (27.1)	0.016		
Dyslipidemia (n, %)	1,460 (70.3)	814 (68.6)	561 (67.2)	650 (66.4)	0.124		
eGFR (mL/min/1.73m^2^)	83.2 ± 18.1	86.4 ± 16.7	87.7 ± 16.5	89.6 ± 16.0	< 0.001		

Abbreviation: eGFR: estimated glomerular filtration rate

Data are presented as mean ± standard deviation or number (percentages)

**Table 2:** Odds ratio with 95% confidence intervals for chronic kidney disease according to physical activity levels

MVPA	CKD prevalence rate (%)	Model 1	Model 2	Model 3
** Inactive** (***n*** = **2**,**077**)	10.83	1 (Reference)	1 (Reference)	1 (Reference)
**1 to 149 min/week** (***n*** = **1**,**187**)	7.25	0.77 (0.59–1.01)	0.78 (0.84–1.03)	0.80 (0.61–1.05)
**150 to 299 min/week** (***n*** = **835**)	7.19	0.84 (0.62–1.15)	0.86 (0.63–1.18)	0.85 (0.62–1.17)
**≥ 300 min/week** (***n*** = **979**)	4.29	**0.52 (0.36–0.74)**	**0.54 (0.38–0.77)**	**0.53 (0.37–0.76)**
***p*** **-trend**		**< 0.001**	**0.001**	**0.001**
**per 1-SD**		**0.74 (0.63–0.89)**	**0.76 (0.64–0.91)**	**0.76 (0.63–0.90)**

MSA	CKD prevalence rate (%)	Model 1	Model 2	Model 3
**0–1 day/week** (***n*** = **4**,**101**)	8.34	1 (Reference)	1 (Reference)	1 (Reference)
**2–3 days/week** (***n*** = **408**)	5.41	0.72 (0.45–1.15)	0.79 (0.49–1.26)	0.82 (0.51–1.32)
**≥ 4 days/week** (***n*** = **569**)	8.61	0.77 (0.56–1.07)	0.85 (0.60–1.19)	0.85 (0.61–1.20)
***p*** **-trend**		0.036	0.200	0.224

Abbreviations: MVPA, moderate-to-vigorous physical activity; MSA, muscle strengthening activity

Model 1: Adjusted for age and sex

Model 2: Model 1 + current smoking status, alcohol consumption, education level, income level, systolic blood pressure, BMI, and MSA in models in which MVPA was exposed or MVPA in models in which MSA was exposed

Model 3: Model 2 + diabetes and dyslipidemia

We found that groups with higher levels of MVPA had a lower OR for CKD compared to the inactive group after adjusting for age, sex, current smoking status, alcohol consumption, education level, income level, SBP, BMI, MSA, diabetes, and dyslipidemia. However, MVPA was associated with the most significant increase in OR when performed for ≥ 300 min/week (OR: 0.53; 95% CI: 0.37–0.76). Moreover, the OR for CKD per SD increase in PA was 0.76 (95% CI: 0.63–0.90) after adjusting for potential confounders in model 3. In contrast, no significant association was found between MSA and the prevalence of CKD.

**Fig. 2:**
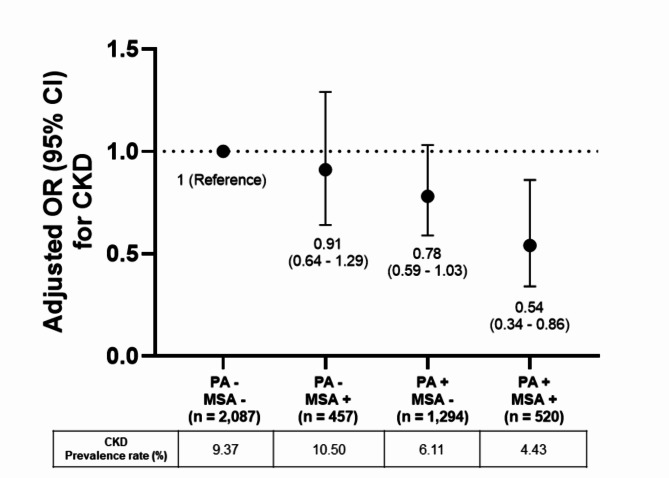
Joint associations of physical activity and muscle-strengthening activity with chronic kidney disease odds ratios. Adjusted for age, sex, current smoking status, alcohol consumption, education level, income level, systolic blood pressure, body mass index, diabetes, and dyslipidemia. Data are expressed as odds ratios with 95% confidence intervals. Physical activity was defined as meeting or not meeting the criteria of ≥ 150 min of moderate-to-vigorous physical activity weekly, with a + or – for meeting or not meeting the guidelines, respectively. Muscle-strengthening activity was defined as practicing ≥ 2 days/week, with a + and - for meeting or not meeting the guidelines, respectively. OR, odds ratio. CKD, chronic kidney disease. PA, physical activity. MSA, muscle-strengthening activity

Figure 2 shows the results of a study that investigated the joint association of aerobic PA and MSA with CKD. After adjusting for all confounding variables, the OR for CKD was 0.91 (95% CI: 0.64–1.29) for the group with MSA at or above recommended levels but aerobic PA level below recommended levels, which was not statistically significant. The OR for CKD was 0.78 (95% CI: 0.59–1.03) in the group with aerobic PA levels at or above the recommended levels but MSA levels below the recommended levels, which were not statistically significant. Finally, the OR for CKD was 0.54 (95% CI: 0.34–0.86) in the group with aerobic PA and MSA levels at or above recommended levels, which was significantly lower than the reference group but was not significantly different from the group with aerobic PA level at or above recommended levels, but MSA level below recommended levels.

## Discussion

We conducted a nationwide study to investigate the association between aerobic PA, MSA levels, and CKD in individuals with hypertension. The highest aerobic PA levels were associated with a lower OR for CKD, whereas MSA levels were not. Particularly, we observed a significantly low OR for CKD in participants who performed ≥ 300 min of MVPA weekly, which is higher than the current aerobic PA guideline recommendations. Moreover, participants who met the aerobic PA and MSA guidelines had a lower OR for CKD than those who met only either guideline. Additionally, we explored the relationship between increased MET levels and increased eGFR values in patients with aerobic PA.

Several studies have investigated the association between PA and CKD [21]. Most observational cross-sectional studies have shown that higher aerobic PA levels are associated with lower CKD prevalence, which is consistent with these study findings [22, 23]. Furthermore, Lin et al. found that antihypertensive medications in patients with diabetes significantly increased the risk of CKD, suggesting that hypertension is a strong risk factor for CKD progression [24]. A study that intervened with a program that included MPA, MSA, and flexibility exercises showed a significant and slow decrease in eGFR-cystatin C levels at the 2-year follow-up [25]. We analyzed the data by dividing the participants into three groups based on their aerobic PA levels. We found that highly active participants with hypertension (MVPA ≥ 300 min/week) had a significantly lower OR of CKD than inactive participants. This suggests that participants with hypertension should be more active than the minimum level recommended by the aerobic PA guidelines to compensate for the risk of CKD. While a study reported that strength training did not significantly improve renal function in hemodialysis patients, such as by reducing albumin levels [26], another study suggested that strength training may positively affect the glomerular basement membrane, as patients who underwent 12 months of strength training had a significant decrease in albuminuria [27]. Furthermore, an animal study on resistance exercise highlighted the importance of the renal protein kinase B (Akt) and mammalian target of rapamycin (mTOR) pathways in CKD, suggesting that resistance exercise is an effective strategy to reduce CKD-related complications [28]. Another study found that muscle weakness in hemodialysis patients was strongly associated with an increased risk of mortality [29], presumably because of its negative impact on the cardiovascular system [30]. Knowingly, this is the first study with a sufficient sample size to investigate the association between aerobic PA, MSA levels, and CKD in individuals with hypertension. Therefore, further research is needed to investigate the relationship between aerobic PA, MSA, and CKD using objective measurements.

Previously, resistance training was not significantly associated with cardiovascular disease (CVD) mortality. However, the highest risk of CVD mortality was observed in the group that did not meet the guidelines for both types of exercise [31]. Moreover, a meta-analysis of experimental studies that combined aerobic PA and resistance training interventions found that exercise interventions delayed the progression of CKD [32]. Therefore, our study indicated that individuals with hypertension who met the recommended guidelines for aerobic and resistance exercises showed the lowest association with CKD, emphasizing the importance of adherence to both types of PA.

The mechanisms by which aerobic PA and combined exercise may be associated with the prevalence of CKD can be considered in multiple ways. The main mechanism of exercise effects in CKD appears to be the attenuation of chronic systemic inflammation, as evidenced by the decreased inflammatory features of immune cells and cytokine secretion [33]. Additionally, exercise-induced Akt phosphorylation in skeletal muscles, either directly or through increased insulin-like growth factor 1 (IGF1) expression, may contribute to enhanced insulin signaling and sensitivity, ultimately leading to increased skeletal muscle mass [33]. Some studies have suggested that aerobic PA and strength training improve blood flow, regulate blood pressure [34], and enhance insulin sensitivity [35]. This improvement in renal blood flow can increase oxygen supply in kidney cells, strengthen vascular walls, and enhance waste removal, potentially promoting kidney tissue regeneration [13]. Although the beneficial effects of aerobic PA and combined exercise on CKD are evident, the potential for reverse causation must be considered. Higher PA levels may be associated with a lower prevalence of CKD owing to improved overall health and renal function [36]. Conversely, individuals with mild CKD may naturally engage in more PA than those with advanced disease stages, potentially confounding this association. Therefore, longitudinal studies are crucial to determine the direction of these relationships and ascertain whether increased PA directly influences CKD outcomes or whether CKD progression inherently restricts PA levels.

The strength of this study lies in its use of national population-based data. To our knowledge, no existing study has explored the association between CKD and resistance exercise frequency in individuals with hypertension. However, this study had several limitations. First, the aerobic PA and MSA data were self-reported, with resistance exercise measured only by the frequency (days/week) of performance. Therefore, recall and social desirability bias are possible; however, the questionnaire has been validated for Koreans and provides information on specific individual activity patterns and times [15]. Second, although the study used national population-based data, potential confounders, such as CVD and frailty, should be considered in the relationships between aerobic PA, MSA, and CKD. Third, CKD was assessed using a single-point eGFR value based on creatinine levels. Therefore, the prevalence of CKD could not be confirmed by observing potential transient changes in creatinine. Finally, this was a cross-sectional study, which limited the ability to establish causality. Future studies should use a cohort design with long-term follow-up to investigate causal relationships.

## Conclusion

In participants with hypertension, we found that aerobic PA and MSA, particularly those exceeding 300 minutes of MVPA per week, were associated with reduced CKD prevalence. Although MSA alone did not show a significant effect, the combination of aerobic PA and MSA proved to be most effective in reducing CKD risk in this group. These results underscore the importance of adhering to PA guidelines to lower the risk of CKD in individuals with hypertension.

## Data Availability

Not applicable.

## Abbreviations

CKD: Chronic kidney disease
WHO: World Health Organization
MVPA: Moderate-to-vigorous physical activity
MSA: Muscle-strengthening activity
KNHANES: Korea National Health and Nutritional Examination Survey
IRB: Institutional Review Board
GPAQ: Global Physical Activity Questionnaire
MPA: Moderate physical activity
MET: Metabolic equivalent of task: VPA: Vigorous physical activity
eGFR: Estimated glomerular filtration rate
BMI: Body mass index
ORs: Odds ratios
CIs: Confidence intervals
SBP: Systolic blood pressure
CVD: Cardiovascular disease
Akt: Renal protein kinase B
mTOR: Mammalian target of rapamycin
IGF1: Insulin-like growth factor 1
